# CircHIPK3 relieves vascular calcification via mediating SIRT1/PGC-1α/MFN2 pathway by interacting with FUS

**DOI:** 10.1186/s12872-023-03602-3

**Published:** 2023-11-27

**Authors:** Siyi Feng, Youfei Qi, Zhanxiang Xiao, Hao Chen, Sahua Liu, Haimei Luo, Hongfei Wu, Wenbo Zhang

**Affiliations:** 1 Department of Ultrasound Medicine, Hainan General Hospital, Hainan Affiliated Hospital of Hainan Medical University, Haikou, 570311 Hainan Province China; 2 Department of Vascular Surgery, Hainan General Hospital, Hainan Affiliated Hospital of Hainan Medical University, No.19, Xiuhua Road, Haikou, 570311 Hainan Province China

**Keywords:** CircHIPK3, Calcification, FUS, SIRT1/PGC-1α signaling

## Abstract

**Background:**

Circular RNAs (circRNAs) have been reported to regulate the biological processes of human diseases. CircHIPK3 has been implicated in vascular calcification, but the downstream regulatory mechanisms remain unclear. Our study aimed to understand the regulatory function of circHIPK3 in vascular calcification.

**Methods:**

CircHIPK3 expression in atherosclerosis (AS) serum samples and vascular smooth muscle cells (VSMCs) calcification model was assessed by quantitative real-time polymerase chain reaction (qRT-PCR). The binding relationships between fused in sarcoma (FUS) and circHIPK3 or sirtuin 1 (SIRT1) were verified by RNA immunoprecipitation (RIP) assay and RNA pull-down assays. Alkaline phosphatase (ALP) activity and alizarin red staining assays were performed to evaluate the biological effect of β-glycerophosphate (β-GP) and circHIPK3 on calcium deposition. qRT-PCR and western blot assays were used to examine the effect of β-GP, circHIPK3, SIRT1, mitofusin 2 (MFN2), and peroxisome proliferator-activated receptor gamma coactivator 1-alpha (PGC-1α) on VSMCs calcification and the expression of calcification-related proteins.

**Results:**

In AS serum samples and VSMCs calcification model, the expression of circHIPK3 was significantly reduced. CircHIPK3 overexpression inhibited ALP activity and calcium deposition in β-GP-induced VSMCs. Moreover, circHIPK3 could recruit FUS to further stabilize SIRT1 mRNA. CircHIPK3 promoted MFN2 expression to alleviate VSMCs calcification via activating SIRT1/PGC-1α signaling.

**Conclusion:**

The positive regulation of circHIPK3/FUS/SIRT1/PGC-1α/MFN2 signaling pathway contributed to the alleviate VSMCs calcification, revealing a novel regulatory axis for vascular calcification.

**Supplementary Information:**

The online version contains supplementary material available at 10.1186/s12872-023-03602-3.

## Introduction

Vascular calcification is considered a pivotal factor to result in various diseases such as atherosclerosis (AS), cerebrovascular disease, and peripheral vascular disease [1, 2]. The characterization of vascular calcification contained the formation of abnormal calcium phosphate crystal deposition and aberrant lipid accumulation [3]. There is no ideal approaches to prevent or reverse vascular calcification. Accordingly, understanding the molecular basis of vascular calcification progression may be beneficial to its improvement.

Circular RNAs (circRNAs) are a class of non-coding RNAs with closed-loop structure, and have gradually regarded as critical players in many cancers and diseases. Recently, extensive evidence has shown that circRNAs are important participants in vascular calcification progression [4]. Previous studies suggested that circSMOc1-2 might be a key regulator of vascular calcification, and its overexpression could prevent vascular calcification by reducing calcium deposition [5]. Besides, circSamd4 was considered to be a biomarker for vascular calcification diagnosis, which silencing had been confirmed to promote vascular calcification progression [6, 7]. CircHIPK3 is derived from exon2 of the HIPK3 gene, and has been found to be differentially expressed in AS model [8]. Studies had shown that circHIPK3 might contribute to vascular smooth muscle cells (VSMCs) proliferation [9]. Additionally, recently study suggested that circHIPK3 could reduce cell mineralization and calcium content to alleviate vascular calcification process [10]. Therefore, circHIPK3 may play an important role in the process of vascular calcification, and its role and new molecular mechanism are worthy of further investigation.

Usually, circRNAs can bind to RNA binding proteins (RBPs) to regulate gene expression at the transcriptional level. Fused in sarcoma (FUS), an RNA binding protein, is reported to bind to non-coding RNAs to further stabilize the mRNA of the downstream target gene. For instance, in acute promyelocytic leukemia, Tang et al. elucidated that lncRNA KCNQ1OT1 recruited FUS to stabilize the mRNA of MAP3K1 [11]. In hepatocellular carcinoma, circ_0004018 was reported to recruit FUS to stabilize TIMP2 expression [12]. Here, we found that circHIPK3 could bind to FUS through bioinformatics prediction (starBase: https://starbase.sysu.edu.cn/index.php) [13]. However, the regulatory effects of between circHIPK3 and FUS in the VSMCs calcification still largely unknown.

Sirtuin 1 (SIRT1), a nicotinamide adenine dinucleotide-dependent histone deacetylase, is reported to participate in cell metabolism, pathophysiology of aging differentiation, and DNA damage [14–17]. SIRT1 is an attractive candidate to mediate vascular calcification by regulating multifarious molecules, including Wnt signaling and RUNX family transcription factor 2 (RUNX2) signaling [18, 19]. In our results, the prediction results of starBase showed that SIRT1 could bind to FUS. Therefore, we speculated that circHIPK3 could stabilize SIRT1 by recruiting FUS.

In this work, we proposed to understand the biological role of circHIPK3 in vascular calcification. Additionally, we verified the existence of circHIPK3/FUS/SIRT1 axis. More importantly, its specific regulatory mechanism was further researched.

## Materials and methods

### AS and healthy samples

A total of 28 AS patients and 28 healthy normal controls in this study were obtained from the Hainan General Hospital, Hainan Affiliated Hospital of Hainan Medical University. The serum samples were collected and stored in -80 °C. The diagnosis of patients with AS was determined on the basis of carotid intima-media thickness (CIMT) of the common carotid artery (CIMT ≥ 1.0 mm) combination with chest radiography, carotid plaques and the actual pathological indicators of the patient, and patients with other clinical conditions were excluded. The clinical characteristics of AS patients and healthy normal controls are listed in Table 1. The work was agreed by the Ethical Committee of the Hainan General Hospital, Hainan Affiliated Hospital of Hainan Medical University.

**Table 1:** The clinical characteristics of AS patients and healthy normal controls

Characteristics	AS (n = 28)	Health (n = 28)	*P* value
Age, years	51.2 ± 10.03	53.5 ± 8.82	0.1762
Sex, male/female	16/12	15/13	0.7881
History of smoking, cases	12	11	0.7859
History of drinking, cases	10	8	0.5671
HDL-C, mmol/L	1.03 ± 0.21	1.01 ± 0.18	0.7035
LDL-C, mmol/L	3.14 ± 0.15	3.53 ± 0.17	< 0.0001
Triglyceride, mmol/L	1.4 ± 0.5	1.2 ± 0.6	0.1811
Total cholesterol, mmol/L	5.2 ± 0.84	4.6 ± 1.3	0.0423
Body mass index (kg/m^2^)	23.12 ± 5.22	21.14 ± 3.64	0.5299
Diabetes, cases	10	6	0.2367
Hypertension, cases	21	7	0.0002

HDL-C, high-density lipoprotein cholesterol; LDL-C, low-density lipoprotein

cholesterol; *P*: AS vs. Health

### Cell culture and treatment

VSMCs were purchased from American Type Culture Collection (Manassas, VA, USA) and cultured in Dulbecco’s Modified Eagle Medium (11,995,065, Gibco, Grand Island, NY, USA) supplemented with 10% fetal bovine serum (FBS; 10,100,147, Gibco) at 37 °C in 5% CO_2_. For observing the effect of β-glycerophosphate (β-GP) on the expression of circHIPK3, VSMCs were incubated with 10 mM β-GP in different time points for 0, 3, 6, 9, and 12d. For establishing a vascular calcification model, VSMCs were treated with 10 mM β-GP for 12 d as previously described [20].

### Cell transfection

Full-length sequences, including circHIPK3, SIRT1 and mitofusin 2 (MFN2), were amplified and cloned into pcDNA3.1 vector to construct circHIPK3, SIRT1 and MFN2 overexpression vectors. The siRNA negative control (si-NC), siSIRT1 and si-peroxisome proliferator-activated receptor gamma coactivator 1-alpha (PGC-1α) were purchased from GenePharma (Shanghai, China). The corresponding cell transfections were performed using Lipo6000™ Transfection Reagent (C0526, Beyotime Biotechnology, Shanghai, China).

### Alkaline phosphatase (ALP) activity analysis

ALP activities in different groups were measured by ALP Assay Kit (P0321M). Specifically, the cell lysis buffer for Western and IP without inhibitors (P0013Ja) was applied to disrupt the VSMCs, and the supernatant was obtained by centrifuging at 10,000 rpm/min for 10 min. All above reagents were purchased from Beyotime Biotechnology. Finally, the absorbance at 405 nm was measured using a microplate reader, and the ALP activity was calculated according to the definition of enzyme activity.

### Alizarin red staining

As previously described [20], Alizarin red staining was performed to detect the mineralized calcium deposits for VSMCs in different treatments. The VSMCs were rinsed by PBS for three times, fixed with 70% ethanol and stained with alizarin red S solution (1%, pH 4.2) (G1452, Solarbio, Shanghai, China) for 1 h. The VSMCs had been washed 3 times with PBS and mineralized calcium deposits were visualized by microscopy (Leica, Wetzlar, Germany).

### Western blot

The total proteins of VSMCs in different groups were extracted by radioimmunoprecipitation analysis buffer (RIPA, R0010) supplemented with 0.5% phenylmethanesulfonyl fluoride (PMSF, P0100). The protein concentrations in different groups were quantified by the bicinchoninic acid (BCA) protein assay kit (PC0020). All above reagents were purchased from Solarbio. Then, protein samples (20 µg total proteins) were submitted to 10% SDS-polyacrylamide gels and transferred to polyvinylidene difluoride membranes. After treated with 5% nonfat milk in PBS containing 0.05% Tween-20, membranes were hatched with primary antibodies at 4 °C overnight followed by incubated with secondary antibody for 1 h at room temperature. The primary antibodies were purchased from Abcam (Cambridge, MA, USA): anti-RUNX2 (ab76956, 1: 2000), anti-osteoprotegerin (OPG) (ab73400, 1:3000), anti-alpha smooth muscle actin antibody (SMA) (ab265588, 1:2500), anti-smooth muscle protein 22α (SM22α) (ab14106, 1:1000), anti-FUS (ab243880, 1:1000), anti-SIRT1 (ab110304, 1:1000), anti-PGC-1α (ab106814, 1:1000), anti-GAPDH (ab9485, 1:2500) and anti-MFN2 (ab124773, 1:1000). The blots were visualized using an ECL chemiluminescent reagent (P0018FS, Beyotime Biotechnology), and the densitometry of each protein band was quantified by Image J software. The blots were cut prior to hybridization with antibodies, so there are no images showing full length membranes.

### Total RNA extraction and quantitative real-time polymerase chain reaction (qRT-PCR)

The total RNA extraction of tissue samples and cells with different transfections was performed by Total RNA Extraction Reagent (R401-01). The cDNA was obtained according to the protocol of HiScript II (R212-01). PCR amplification was performed using SYBR qPCR Mix (Q711-02/03). All above reagents were purchased from Vazyme (Nanjing, China). CircHIPK3, RUNX2, OPG, SMA, SM22α, SIRT1, PGC-1α and MFN2 expressions were normalized by GAPDH. The primer sequences are displayed in Table 2.

**Table 2:** Primers in this work are listed

Gene	Forward (5’-3’)	Reverse (5’-3’)
circHIPK3	CTCAGCCAGTTAGTGTGGGG	TGTGAGGCCATACCTGTTCTG
SMA	GCTCCCAGGCTAGAGAGCAT	CACCATCACCCCCTGATGTC
SM22α	TGGGCACTACCGTGGAGA	AGGCCAATGACATGCTTTCC
RUNX2	TATGGCACTTCGTCAGGAT	CTTCCATCAGCGTCAACAC
OPG	TCAGTTTGTGGCGAATAA	TGGACCTGGTTACCTATC
SIRT1	GATGAACCGCTTGCTAT	TGAGGGAAGACCCAATAA
PGC-1α	TCACCACCCAAATCCTTA	TGACTCATAGTAATAGCAGGA
MFN2	GCCAGTGCTTCTCCCTCA	GCTGGACCTGGTCATTGTAG
GAPDH	GGGAAACTGTGGCGTGAT	GGGTGTCGCTGTTGAAGT

### ELISA

The concentrations of PGC-1α and MFN2 in cell culture supernatant were detected by PGC-1α ELISA Kit and MFN2 ELISA Kit (Shanghai Jianglai industrial Limited By Share Ltd., Shanghai, China) according to kit instructions.

### RNA immunoprecipitation (RIP) assay

RNA Binding Protein Immunoprecipitation Assay Kit (KT102-01; Saicheng Biotech Co., Ltd, Guangzhou, China) was applied to carry out RIP assay. Briefly, 4 × 10^7^ VSMCs were lysed and then 100 µL of supernatant was collected to incubate with the magnetic beads. The magnetic beads were previously conjugated with anti-FUS antibodies (1:1000; ab243880; Abcam) and control anti-IgG antibodies. The magnetic beads were eluted and the RNA complex was purified to obtain the RNA for qRT-PCR analysis.

### RNA pull-down assay

RNA pull-down assay was performed by RNA pull-down kit (KT103-01; Guangzhou Saicheng Biotech Co., Ltd). In brief, 4 × 10^7^ VSMCs were lysed and centrifuged to collect the supernatant. The magnetic beads (50 µL) were conjugated with biotinylated circHIPK3 or SIRT1 probes and incubated with the supernatant (100 µL) at 4 °C overnight. Next, the beads were eluted from the RNA-protein complex. The FUS protein was detected by western blot assay.

### RNA degradation assay

VSMCs in logarithmic phase were collected and incubated with 0.5 µM actinomycin D (Act D) for 0, 3, 6, 9, and 12 h. After incubation, VSMCs in different groups were collected and lysed to measure the SIRT1 mRNA expression by qRT-PCR.

### Statistical analysis

All experiments were performed in triplicate, with each independent experiment set 3 times to generate an average value. Data are presented as means ± SD. Results were analyzed with one-way ANOVA and *t*-test using GraphPad Prism 7.0 and SPSS 19.0 software. *P* < 0.05 was considered statistically significant (*P < 0.05; **P < 0.01; ***P < 0.001).

## Result

### CircHIPK3 is poorly expressed in AS and vascular calcification model

**Fig. 1:**
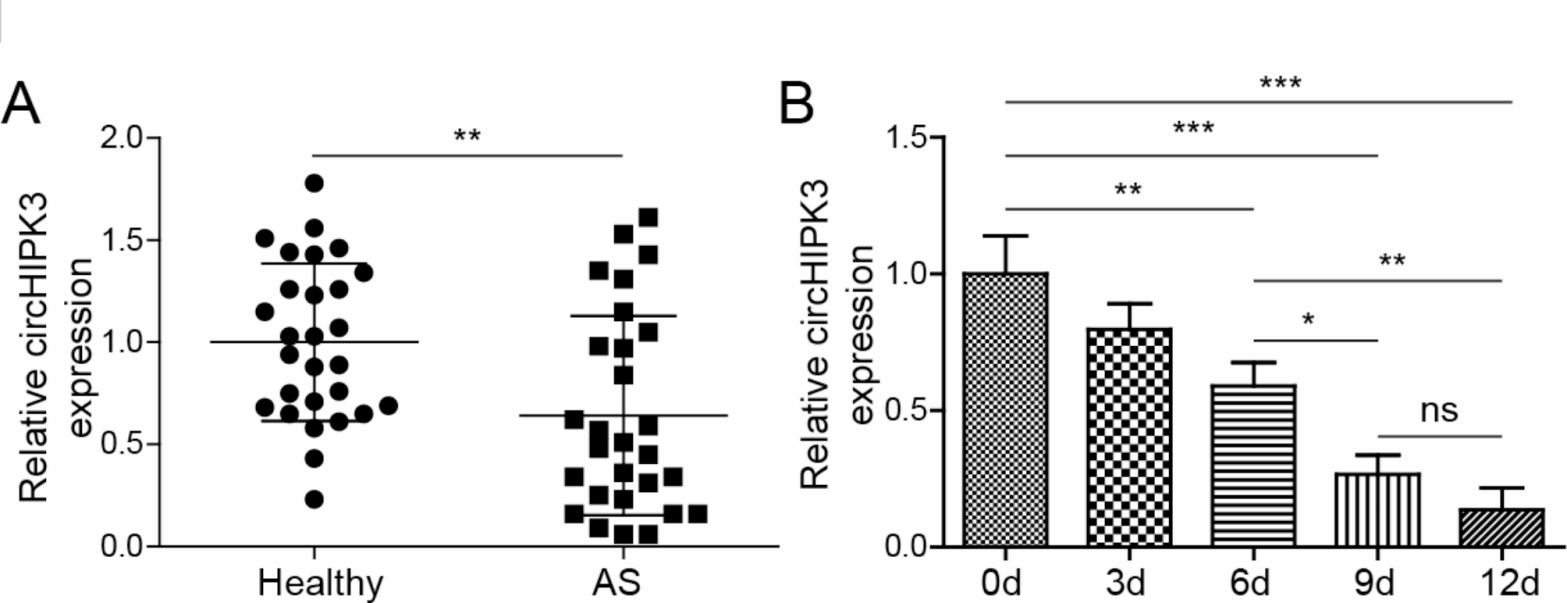
CircHIPK3 expression is down-regulated in AS and β-GP-induced VSMCs. (**A**) Relative expressions of circHIPK3 in AS and heathy serum samples were assessed by qRT-PCR. (**B**) CircHIPK3 expression level was analyzed in VSMCs cultured with 10 mM β-GP for 0, 3, 6, 9 and 12 days. *P < 0.05, **P < 0.01, ***P < 0.001

To research the function of circHIPK3 in AS, its expression was tested in the serum samples of AS patients and healthy normal controls. We found that circHIPK3 expression in AS serum samples was down-regulated compared with healthy samples by qRT-PCR analysis (Fig. 1A). In VSMCs incubated with 10 mM β-GP for 0, 3, 6, 9 and 12 days, circHIPK3 expression was significantly decreased in a time dependent manner (Fig. 1B). Therefore, we choose the stimulation condition (10 mM β-GP for 12 days) for further experiments.

### Overexpression of circHIPK3 ameliorates β-GP-induced calcification

**Fig. 2:**
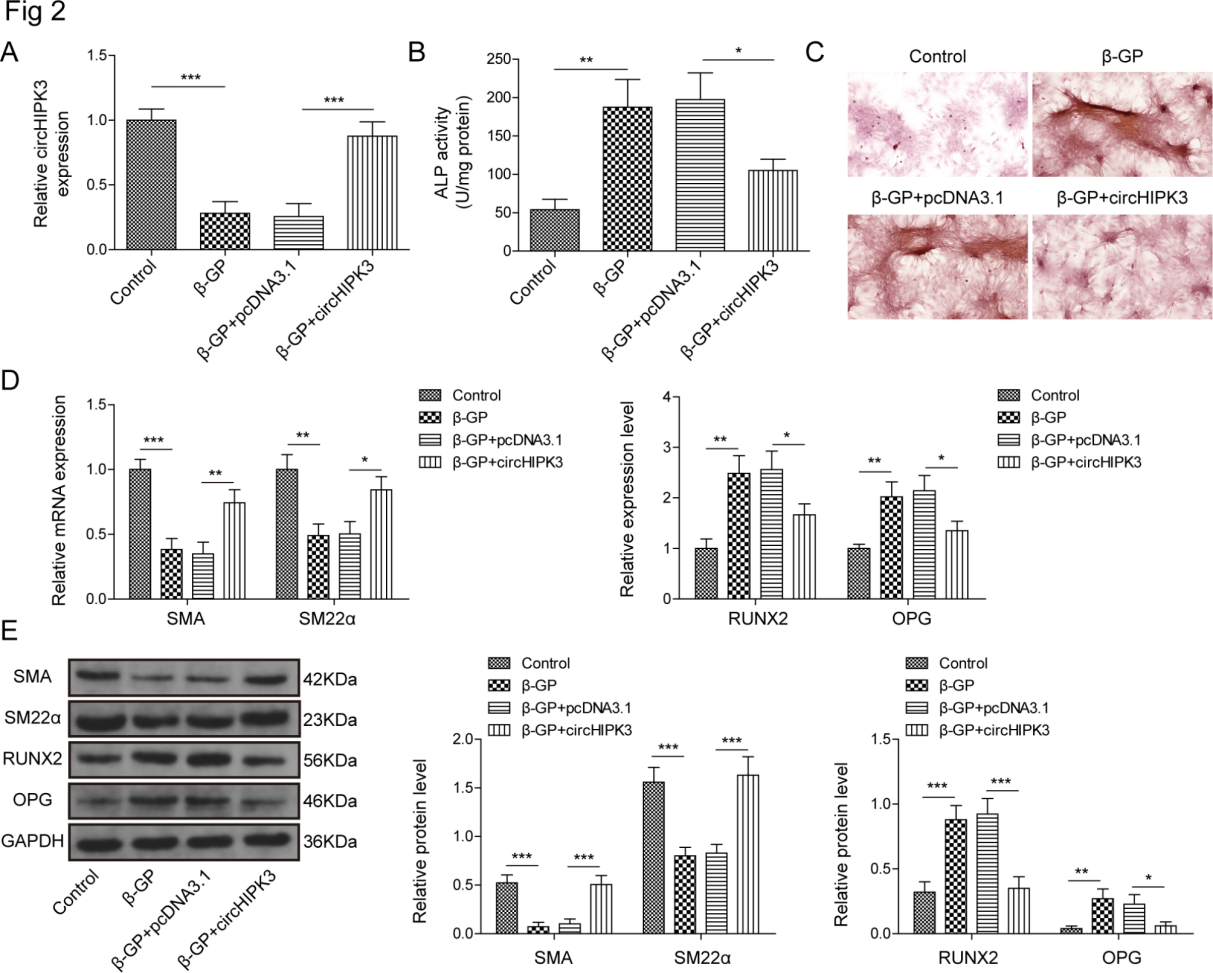
Overexpression of circHIPK3 attenuates β-GP-induced calcification in VSMCs. In control, β-GP (12d), β-GP + pcDNA3.1 and β-GP + circHIPK3 group, (**A**) The expression of circHIPK3 was validated by qRT-PCR analysis. (**B**) ALP activity was examined in all groups. (**C**) Alizarin red S staining of VSMCs was observed in different treatments. qRT-PCR (**D**) and western blot (**E**) analysis of SMA, SM22α, RUNX2 and OPG were performed. *P < 0.05, **P < 0.01, ***P < 0.001

In Figure. S1A, the expression of circHIPK3 was dramatically up-regulated nearly 3-fold in circHIPK3 group compared with control or pcDNA3.1 group. To explore the role of circHIPK3 in β-GP-induced VSMCs calcification, circHIPK3 overexpressed VSMCs were treated with β-GP for 12 days. The qRT-PCR result exhibited that circHIPK3 overexpression vector markedly enhanced circHIPK3 expression suppressed by the stimulation of β-GP (Fig. 2A). Next, ALP activity and alizarin red staining were performed for the identification of VSMCs calcification. ALP activity and extensive mineralized calcium deposits were increased in VSMCs treated with 10 mM β-GP over a time span of 12 days. However, overexpression of circHIPK3 remarkably weakened ALP activity and calcium deposition in β-GP-treated VSMCs (Fig. 2B and C). In addition, β-GP induction reduced the expression of SMA and SM22α and increased the expression of RUNX2 and OPG in VSMCs, while these effects were eliminated by circHIPK3 overexpression (Fig. 2D and E). Collectively, our findings suggest that circHIPK3 overexpression may reduce the β-GP-induced calcification of VSMCs.

### CircHIPK3 binds to FUS to stabilize SIRT1 mRNA

**Fig. 3:**
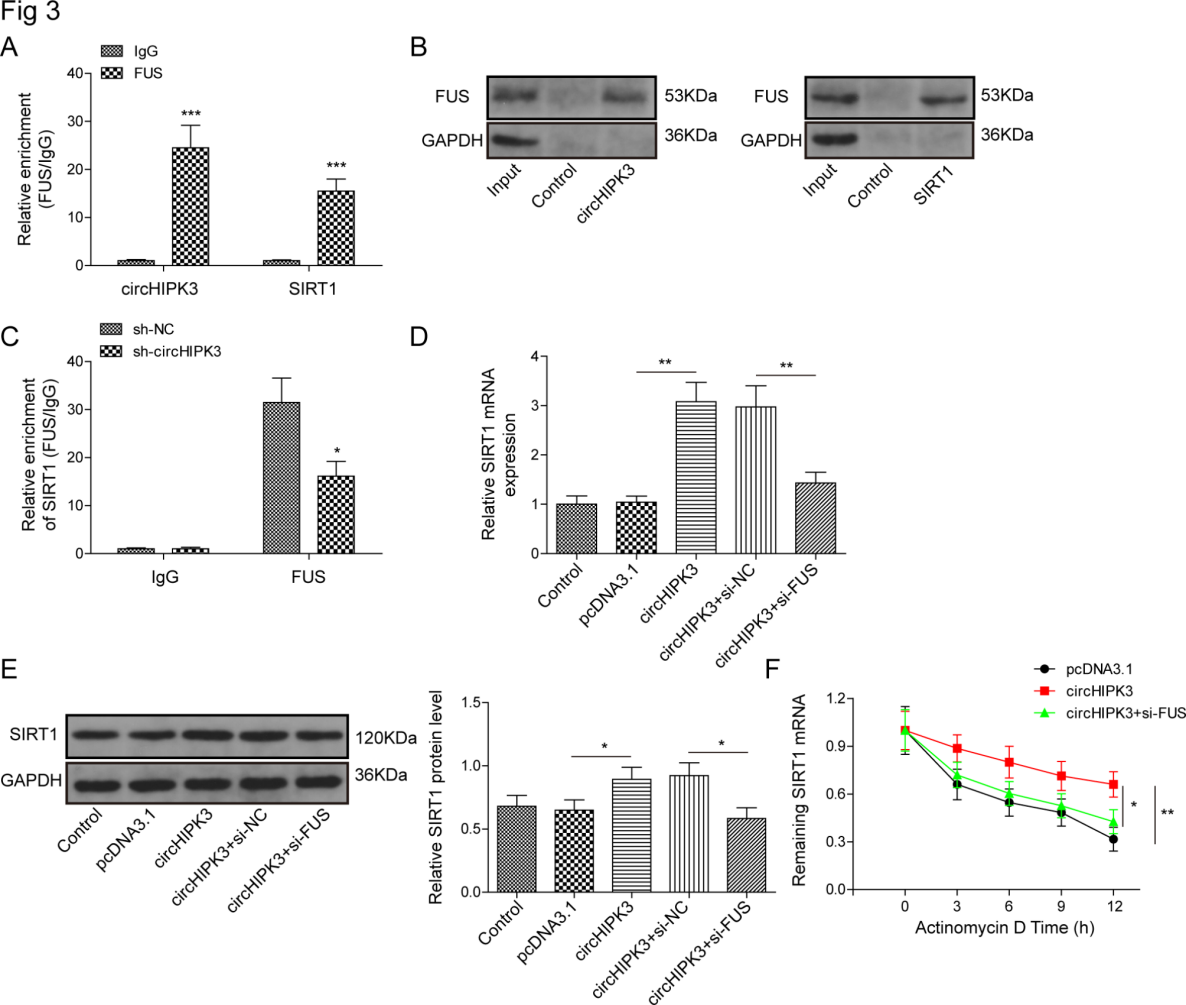
CircHIPK3 interacts with FUS to inhibit SIRT1 mRNA degradation. (**A**) RIP and (**B**) RNA pull-down assays were conducted to confirm the interaction for circHIPK3 and FUS or SIRT1 and FUS. (**C**) The effect of knockdown of circHIPK3 on the relative enrichment of FUS was detected by RIP. The mRNA (**D**) and protein (**E**) expression levels of SIRT1 in VSMCs transfected with pcDNA3.1, circHIPK3 vector, circHIPK3 + si-NC, and circHIPK3 + si-FUS group. (**F**)The stability of SIRT1 mRNA in VSMCs was determined after Act D treatment for 0, 3, 6, 9, and 12 h. *P < 0.05, **P < 0.01, ***P < 0.001

Through starBase (https://starbase.sysu.edu.cn/index.php), we found 6 targets (UPF1, PTBP1, TAF15, FTO, FUS and U2AF2) that could bind to circHIPK3 and SIRT1. In the previous study, we detected the binding of these targets with circHIPK3 and SIRT1 through RIP assay, and found that 5 targets (UPF1, PTBP1, TAF15, FTO and U2AF2) could not simultaneously combine with circHIPK3 and SIRT1 together (Figure. S2). However, FUS could simultaneously bind to both circHIPK3 and SIRT1, which showed that anti-FUS antibodies enriched a prominent higher level of circHIPK3 and SIRT1 (Fig. 3A), so FUS was chosen to our research. RNA pull-down assay exhibited that circHIPK3 or SIRT1 notably enriched FUS proteins (Fig. 3B). The knockdown of circHIPK3 markedly decreased the relative enrichment of FUS (Fig. 3C). Furthermore, depletion of FUS reversed the increased mRNA and protein levels of SIRT1 induced by overexpressing circHIPK3 (Fig. 3D and E). The stability of SIRT1 in all groups was determined after Act D treatment. The knockdown of FUS prominently reduced the high stability of SIRT1 mRNA levels mediated by the overexpression of circHIPK3 (Fig. 3F).

### SIRT1 promotes MFN2 expression to inhibit VSMCs calcification via PGC-1α

**Fig. 4:**
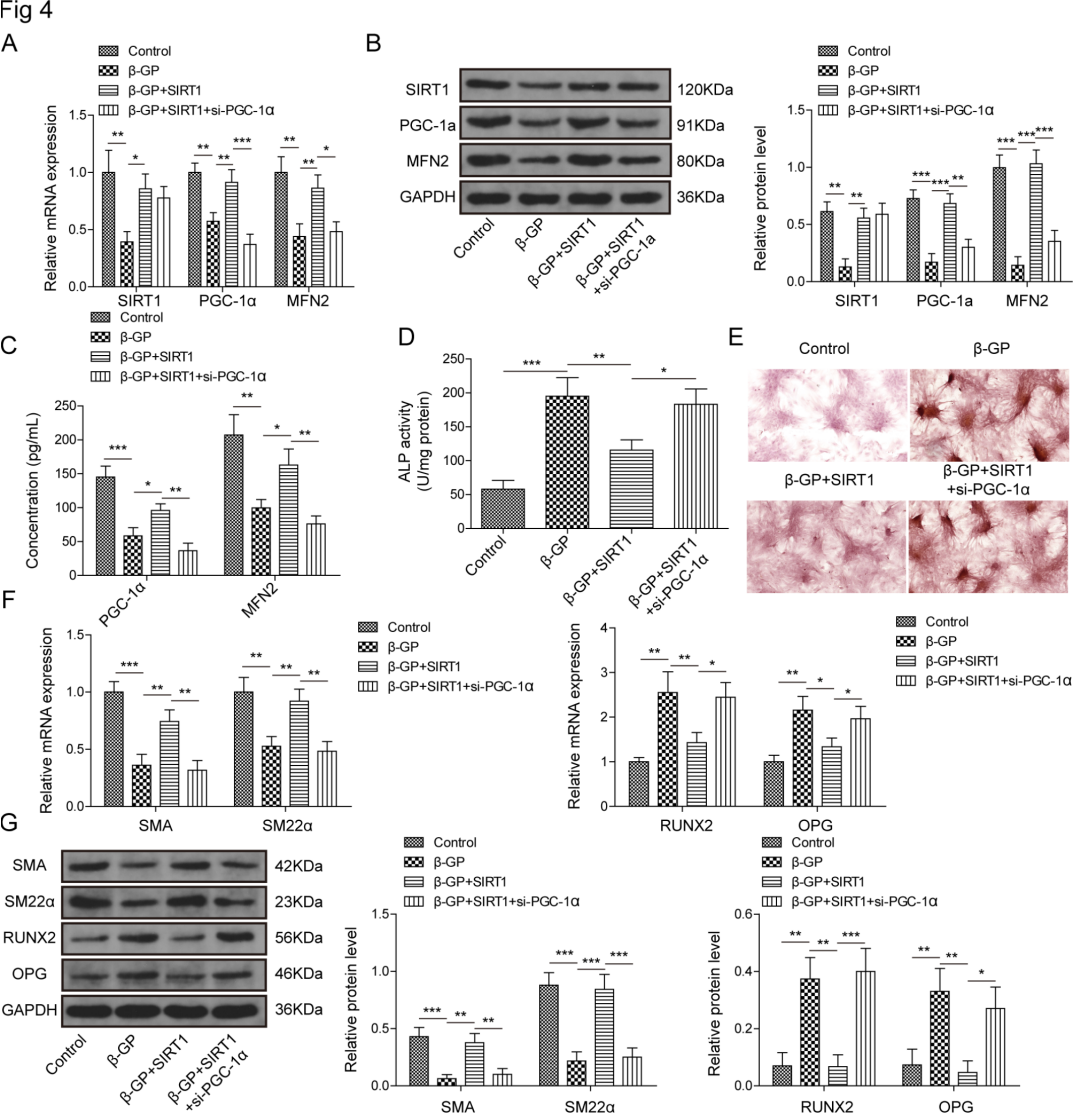
SIRT1 promotes MFN2 expression to mediate VSMCs calcification via PGC-1α. In control, β-GP, β-GP + SIRT1 and β-GP + SIRT1 + si-PGC-1α group, qRT-PCR (**A**) and western blot (**B**) analysis of SIRT1, PGC-1α and MFN2 was performed. (**C**) ELISA was used to detect the concentrations of SIRT1 and PGC-1α. The quantitative analysis of ALP activity (**D**) and alizarin red staining (**E**) was performed in all group. Relative mRNA (**F**) and protein (**G**) levels of SMA, SM22α, RUNX2 and OPG were detected in different groups. *P < 0.05, **P < 0.01, ***P < 0.001

In intestinal epithelial cells, SIRT1/PGC-1α pathway was reported to regulate the autophagy and oxidative damage [21]. The activation the PGC-1α/TERT pathway effectively ameliorated AS [22]. In addition, MFN2 was a downstream target of PGC-1α [23]. The functional relationships were subsequently examined among SIRT1, PGC-1α and MFN2. The qRT-PCR and western blot results showed a significant increase of SIRT1 expression in SIRT1 overexpressing-vector transfection group compared with pcDNA 3.1 group (Figure. S1B). Compared with si-NC group, a noteworthy decrease of PGC-1α was detected in si-PGC-1α group (Figure. S1C). SIRT1 overexpression positively rescued the inhibitory effect of β-GP treatment on the mRNA and protein expressions of SIRT1, PGC-1α and MFN2. Compared with β-GP + SIRT1 group, the mRNA and protein levels of PGC-1α and MFN2 were reduced when PGC-1α was inhibited, while SIRT1 had no significant influence (Fig. 4A and B), suggesting that SIRT1 located in the upstream of PGC-1α and MFN2. Besides, ELISA results suggested that SIRT1 overexpression enhanced the concentrations of PGC-1α and MFN2 in β-GP-treated VSMCs, and this effect could be reversed by PGC-1α knockdown (Fig. 4 C). Next, ALP activity and alizarin red staining assays revealed that the overexpression of SIRT1 retarded the promotion effect on VSMCs calcification by treating the β-GP. ALP activity and calcium deposition were promoted in β-GP + SIRT1 + si-PGC-1α group as compared with β-GP + SIRT1 + si-PGC-1α group (Fig. 4D and E). SIRT1 overexpression enhanced the expression of SMA and SM22-α, while reduced the expression of RUNX2 and OPG in β-GP-induced VSMCs. However, these effects also were reversed by PGC-1α knockdown (Fig. 4F and G). Hence, SIRT1 reduced β-GP-induced calcification in VSMCs via promoting MFN2 expression mediated by PGC-1α.

### CircHIPK3 promotes MFN2 to alleviate VSMCs calcification via SIRT1/PGC-1α

**Fig. 5:**
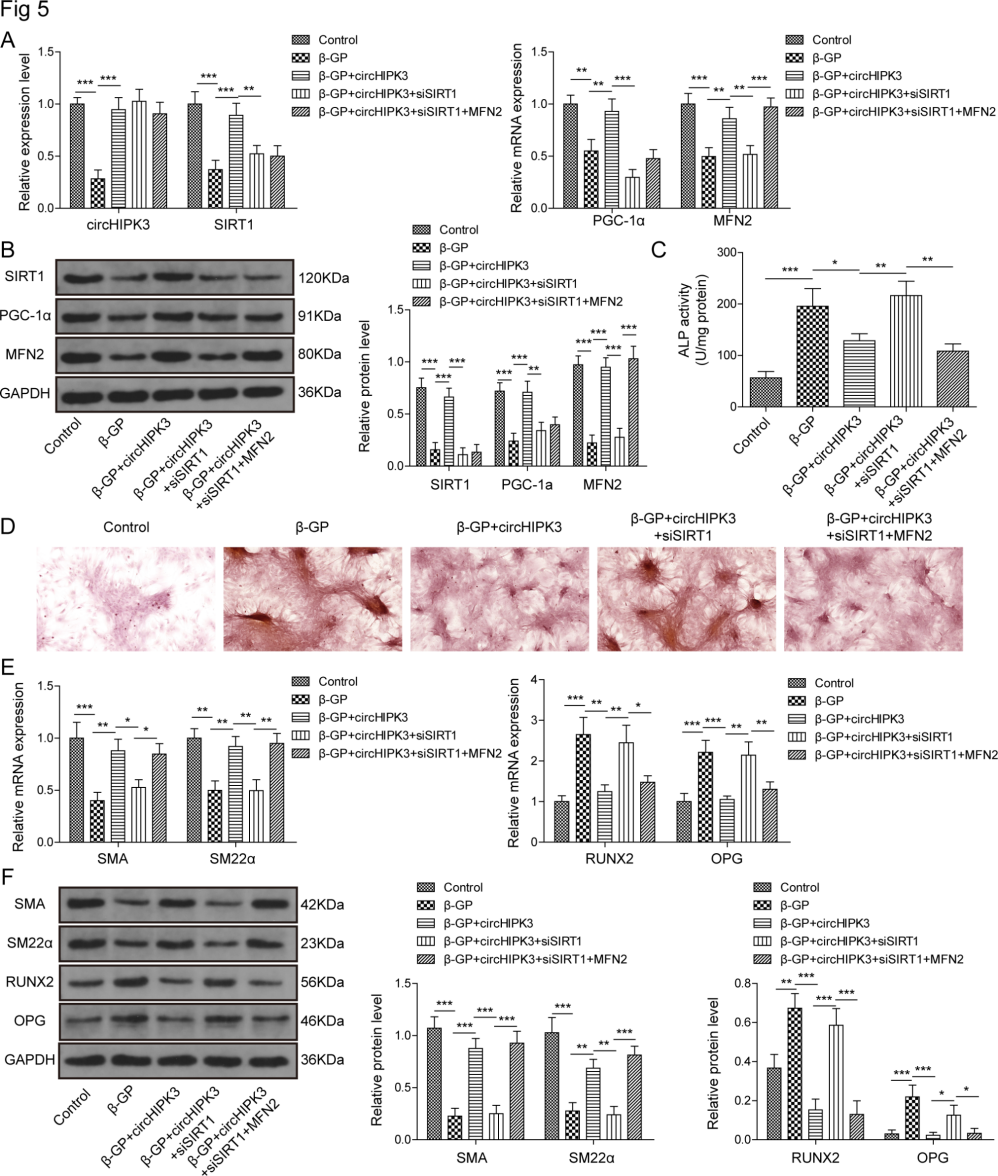
CircHIPK3 promotes MFN2 to change VSMCs calcification via SIRT1/PGC-1α. In control, β-GP, β-GP + circHIPK3, β-GP + circHIPK3 + siSIRT1, and β-GP + circHIPK3 + siSIRT1 + MFN2 group, qRT-PCR (**A**) and western blot (**B**) analysis of SIRT1, PGC-1α and MFN2 was performed. ALP activity (**C**) and alizarin red staining (**D**) were analyzed in all group. SMA, SM22α, RUNX2 and OPG mRNA (**E**) and protein (**F**) expressions were detected in different groups. *P < 0.05, **P < 0.01, ***P < 0.001

**Fig. 6:**
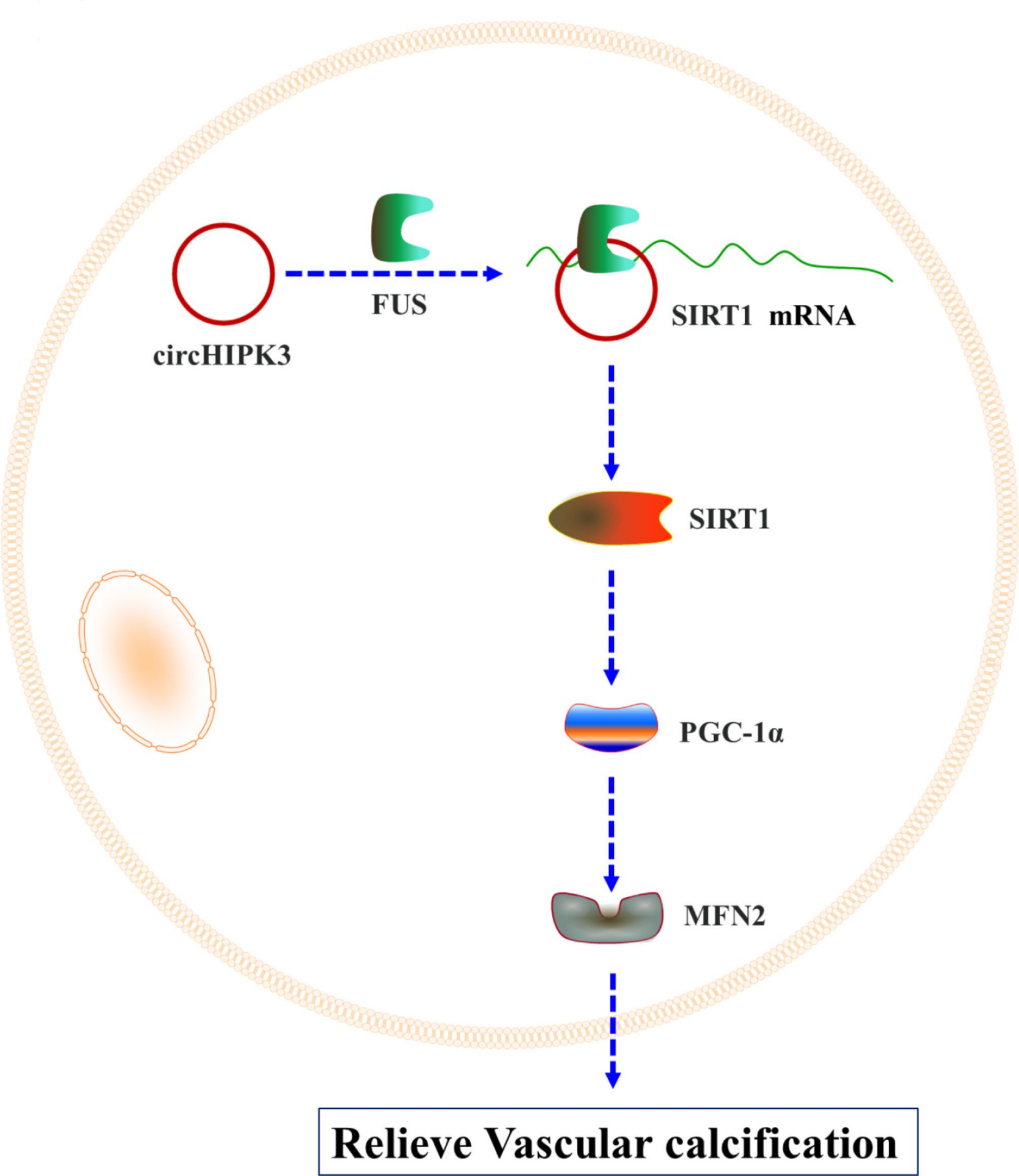
Mechanism diagram of this study. CircHIPK3 stabilized SIRT1 mRNA by interacting with FUS and relieved VSMCs calcification via PGC-1α/MFN2 signaling pathway

In order to explore the roles of circHIPK3/SIRT1 axis in VSMCs calcification, the expression levels of circHIPK3, SIRT1, PGC-1α and MFN2 were tested in control, β-GP, β-GP + circHIPK3, β-GP + circHIPK3 + siSIRT1, and β-GP + circHIPK3 + siSIRT1 + MFN2 group. Compared with si-NC group, a noteworthy decrease of SIRT1 was detected in siSIRT1 group (Figure. S1B). The high mRNA and protein levels of MFN2 were observed in VSMCs transfected with MFN2-overexpressing vector compared with pcDNA 3.1 group (Figure. S1D). As presented, a 12-day stimulation with β-GP, the low expression levels of circHIPK3, SIRT1, PGC-1α and MFN2 were observed. CircHIPK3 overexpression alleviated these reductions for SIRT1, PGC-1α and MFN2. The knockdown of SIRT1 inhibited the expression of PGC-1α and MFN2, and had no significant influence on the expression of circHIPK3 in β-GP + circHIPK3 + siSIRT1 group, suggesting that SIRT1, PGC-1α and MFN2 could be regulated by circHIPK3. In β-GP + circHIPK3 + siSIRT1 + MFN2 group, MFN2 overexpression only restored the mRNA and protein levels of MFN2, indicating that MFN2 was regulated by circHIPK3/SIRT1/PGC-1α axis (Fig. 5A and B). The administration of siSIRT1 in β-GP/circHIPK3-treated cells displayed the enhanced ALP activity and the accumulation of mineralized calcium deposits compared with β-GP + circHIPK3 group, while these effects were attenuated by the co-overexpression of MFN2 (Fig. 5 C and D). Inhibition of SIRT1 reversed the activation effect of circHIPK3 on SMA and SM22-α resulted from co-processing with β-GP and circHIPK3. MFN2 overexpression rescued inhibitory effect caused by β-GP + circHIPK3 + siSIRT1 treatment on the mRNA and protein levels of SMA and SM22-α. And the contrary results were observed in RUNX2 and OPG (Fig. 5E and F). To sum up, our data suggested that circHIPK3 might relieve vascular calcification process through the FUS/SIRT1/PGC-1α/MFN2 axis (Fig. 6).

## Discussion

In this paper, we explored the role and mechanism of circHIPK3 in vascular calcification. Our data demonstrated that the expression level of circHIPK3 was down-regulated in AS and β-GP-induced vascular calcification model. Functional experiments suggested that circHIPK3 overexpression could relieve vascular calcification process. These results provide new evidence for circHIPK3 as a potential target for vascular calcification therapy.

FUS protein belongs to FET family of RNA-binding proteins and involves in the regulation of gene expression. Commonly, FUS is researched as a RBP for non-coding RNAs in oncology and various diseases. On the one hand, FUS binds to circRNAs to further stabilize the mRNA of the downstream target gene. In laryngeal squamous cell cancer, FUS combined with hsa_circ_0006232 to promote the stabilization of EZH2 [24]. Besides, circ0005276 was found to recruit FUS to increase mRNA stability of XIAP, thereby promoting prostate cancer progression [25]. CircFndc3b performed its function to recruit FUS to increase vascular endothelial growth factor-A expression to modulate cardiac repair [26]. Through starBase prediction and further screening, we found that FUS had binding sites with circHIPK3 and SIRT1. Further analysis revealed that circHIPK3 could bind to FUS, and circHIPK3 recruited the FUS to stabilize SIRT1 mRNA.

SIRT1/PGC-1α pathway was reported to involve in a variety of biological processes. In ischemic heart rat model, SIRT1-PGC-1α pathway participated in energy metabolism mediated by Danqi Tablet [23]. 20-HETE inhibited the SIRT1/PGC-1α signaling to accelerate the neuronal apoptosis [27]. The activation of SIRT1/PGC-1α pathway relieved the cellular injury in the proximal tubule induced by kidney ischemia-reperfusion [28]. PGC-1α was reported as an upstream mediator of MFN2 in alcoholic liver disease and liver ischaemia-reperfusion injury [29, 30]. However, the relationship of SIRT1, PGC-1α and MFN2 in the process of VSMCs calcification had not explored. In our work, we found that SIRT1 promoted the expression of PGC-1α and MFN2. Besides, we confirmed that SIRT1 overexpression inhibited VSMCs calcification, which was consistent with previous results [31]. Further knockdown of PGC-1α on the basis of overexpression of SIRT1 resulted in a decrease in the expression of MFN2 and aggravated the VSMCs calcification, confirming that SITR1 repressed VSMCs calcification by increasing MFN2 expression via regulating PGC-1α. In addition, SIRT1 knockdown also reversed the inhibitory effect of circHIPK3 on VSMCs calcification, and these effects were eliminated by MFN2 overexpression. Moreover, overexpression of MFN2 had no significant influence on the expression of SIRT1 and PGC-1α, but mitigated the degree of VSMCs calcification in β-GP + circHIPK3 + siSIRT1 group, further suggesting that PGC-1α was upstream of MFN2. Therefore, we speculated that circHIPK3 regulated SIRT1/PGC-1α/MFN2 axis to alleviate VSMCs calcification by recruiting FUS.

Of course, there are some limitations to our study. Vascular calcification is a highly complex process, and a single circRNA is not sufficient. Our study only explored the underlying molecular mechanism affecting the process of vascular calcification at the basic level, and revealed that circHIPK3 may participate in the process of vascular calcification through the FUS/SIRT1/PGC-1α/MFN2 axis. More circRNAs or pathways involved in vascular calcification need to be further explored. In addition, we have not explored the existence of circHIPK3/FUS/SIRT1/PGC-1α/MFN2 axis by more animal experiments and clinical level verifications. This study urgently needs further investigation to reveal the underlying mechanism of circHIPK3 in VSMCs calcification.

In summary, our present results revealed the inhibitory effect and mechanism of circHIPK3 in VSMCs calcification, and confirmed the existence of circHIPK3/FUS/SIRT1/PGC-1α/MFN2 axis. Our study may provide a novel strategy for the therapy of vascular calcification-related diseases.

### Electronic supplementary material

Below is the link to the electronic supplementary material.

Supplementary Material 1

Supplementary Material 2

Supplementary Material 3

Supplementary Material 4

## Data Availability

The datasets used or analyzed during the current study are available from the corresponding author on reasonable request.

## Abbreviations

AS: Atherosclerosis
circRNAs: Circular RNAs
β-GP: β-glycerophosphate
FUS: Fused in sarcoma
MFN2: Mitofusin 2
OPG: Osteoprotegerin
PGC-1α: Peroxisome proliferator-activated receptor-gamma coactivator-1alpha
ALP: Alkaline phosphatase
RBP: RNA binding protein
RUNX2: Runt-related protein 2
SIRT1: Sirtuin 1
SMA: Smooth muscle actin
SM22α: Smooth muscle protein 22α
VSMCs: Vascular smooth muscle cells
