# Supplementary figures and images for: CircHIPK3 relieves vascular calcification via mediating SIRT1/PGC-1α/MFN2 pathway by interacting with FUS
Source: BMC Cardiovasc Disord. 2023 Nov 27;23:583. doi: 10.1186/s12872-023-03602-3 (PMC10683355; doi:10.1186/s12872-023-03602-3)

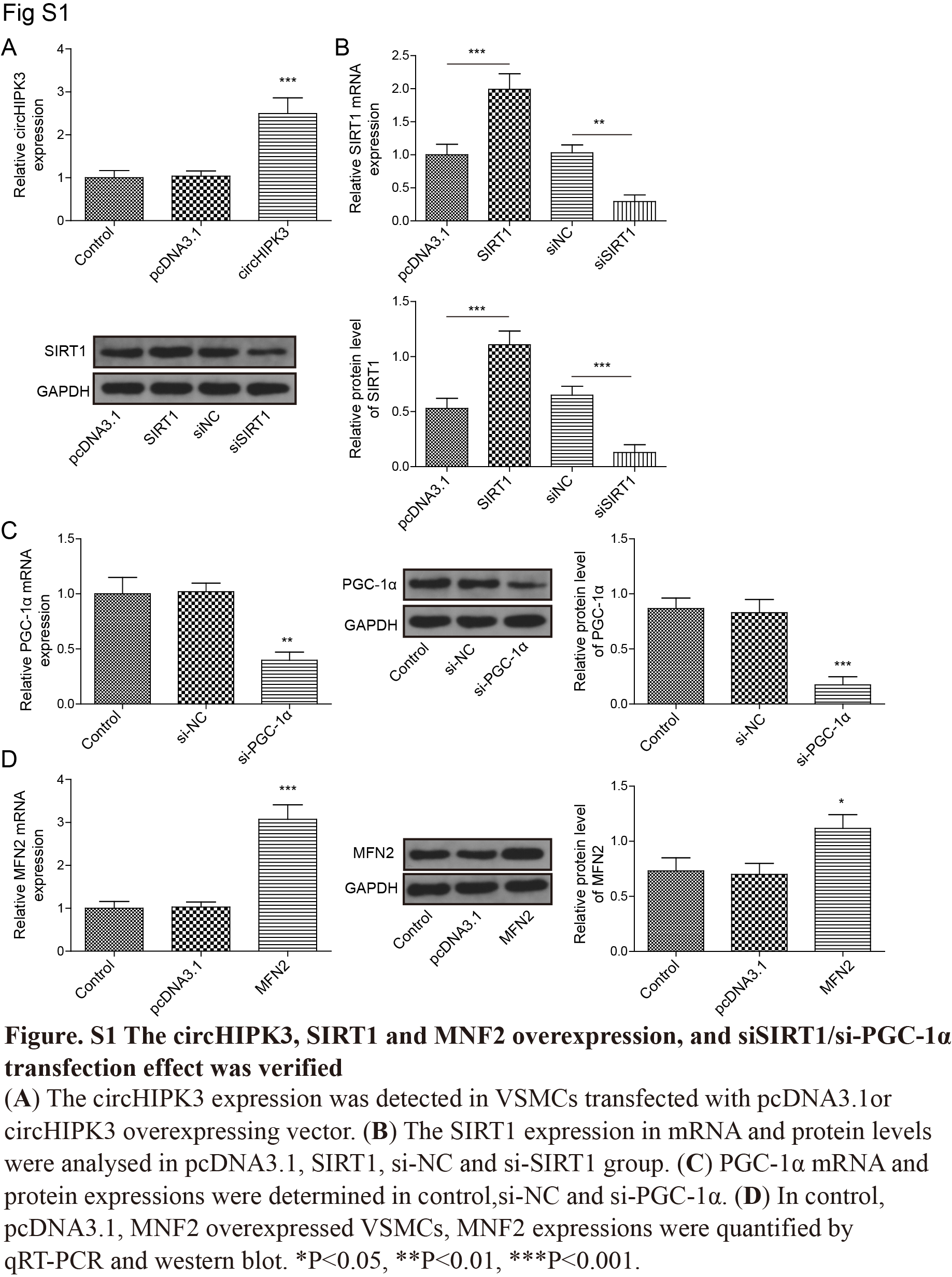

Supplement: Supplementary file 1 — Supplementary Material 1 [file 12872_2023_3602_MOESM1_ESM.png]

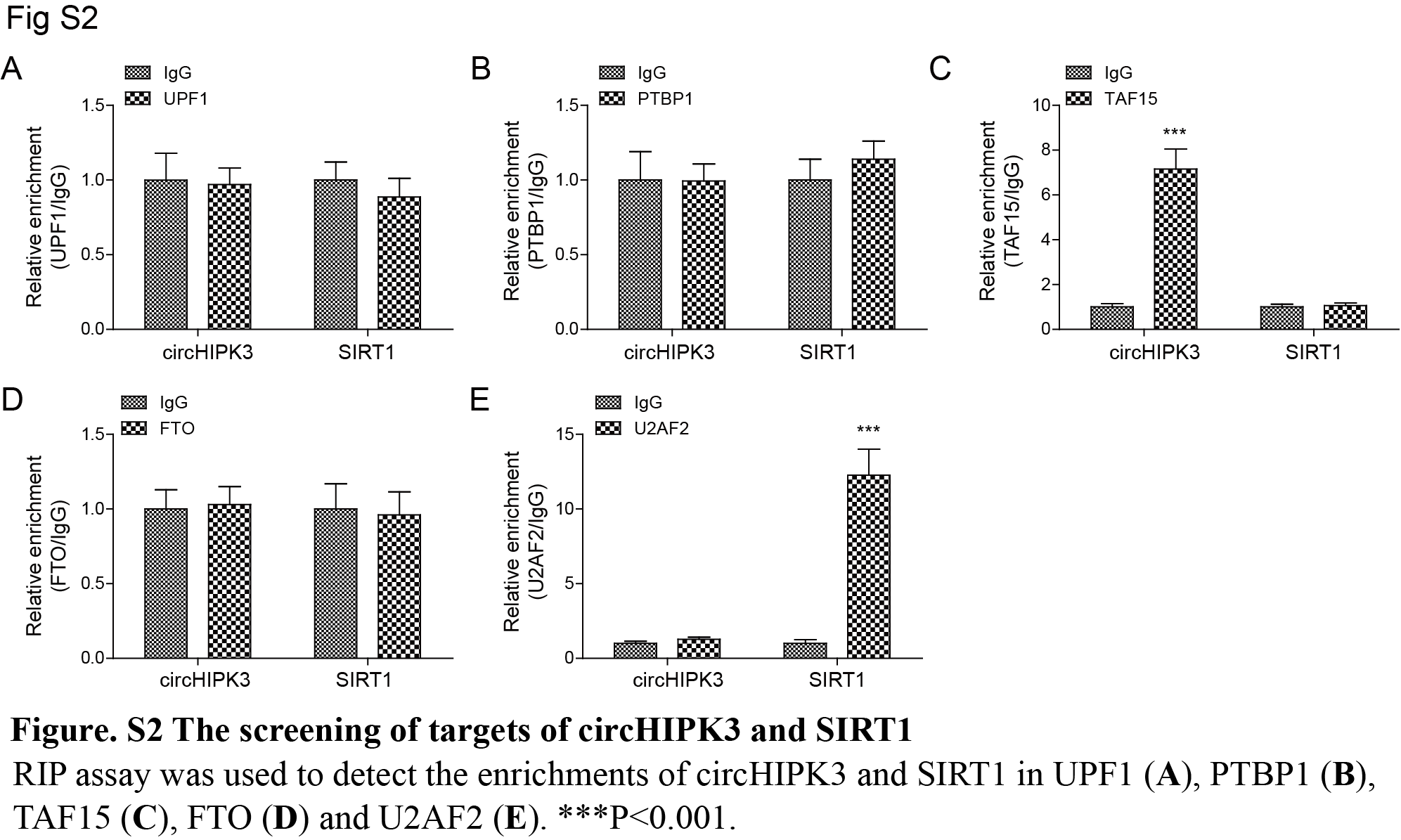

Supplement: Supplementary file 2 — Supplementary Material 2 [file 12872_2023_3602_MOESM2_ESM.png]

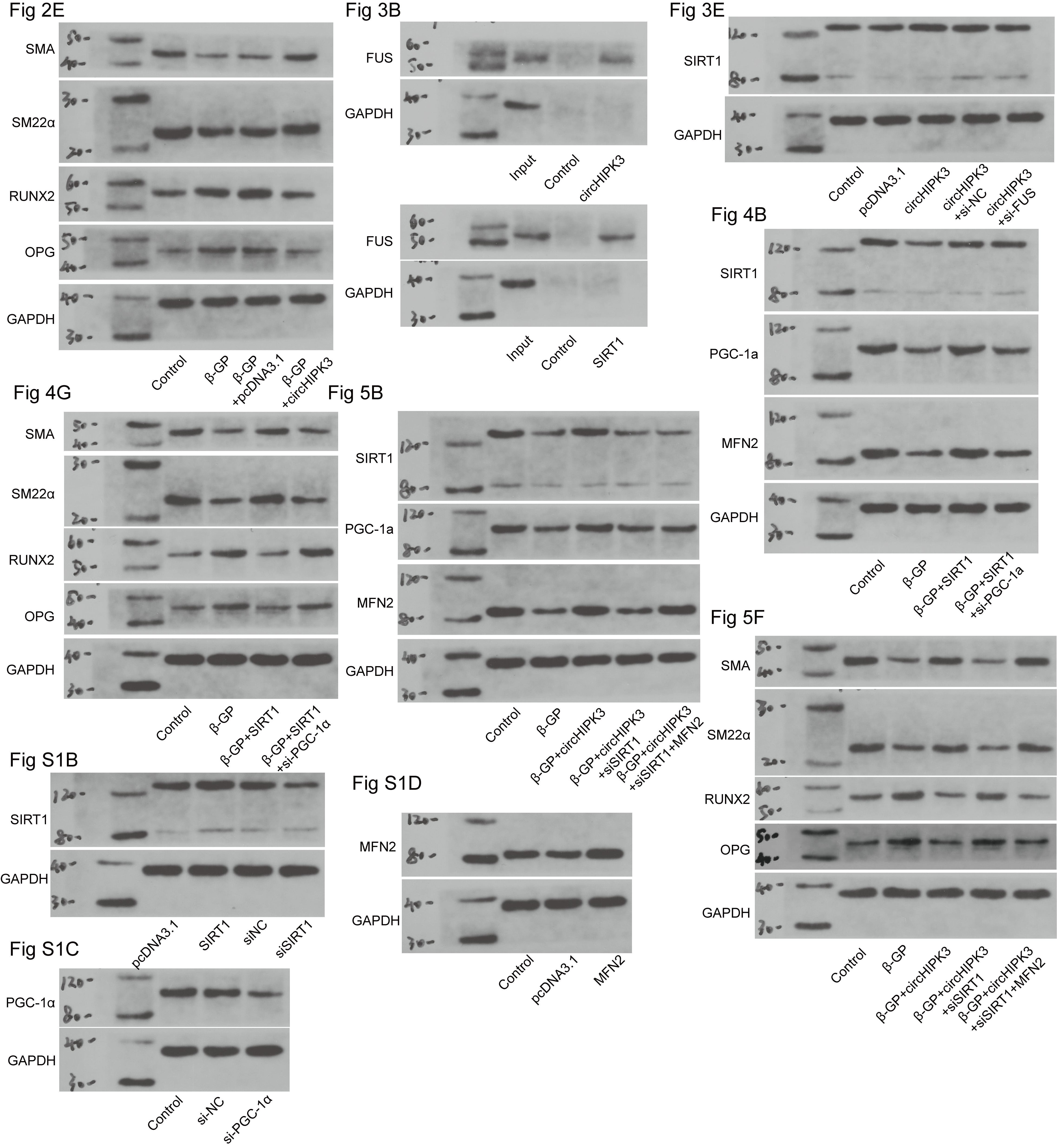

Supplement: Supplementary file 3 — Supplementary Material 3 [file 12872_2023_3602_MOESM3_ESM.png]

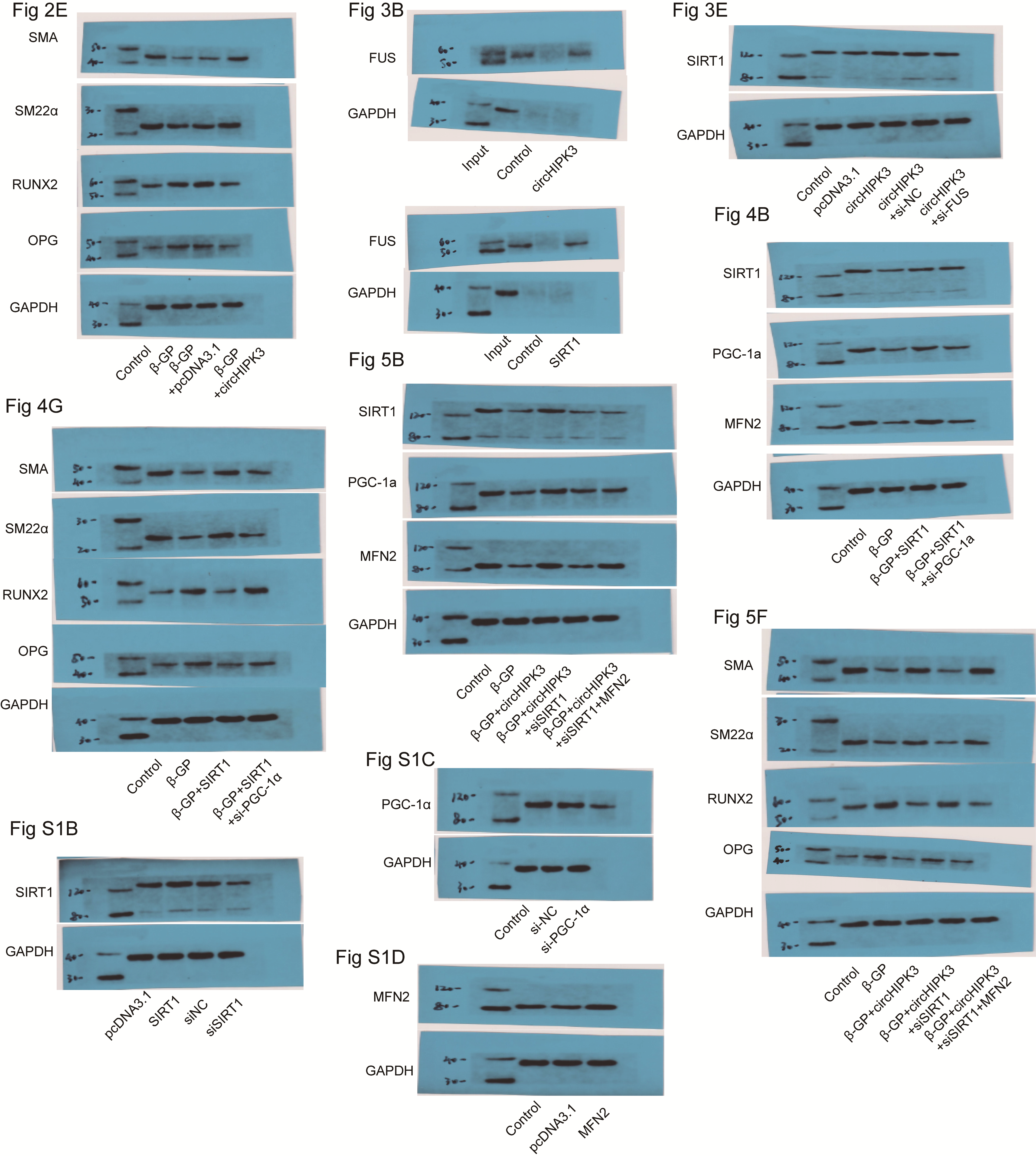

Supplement: Supplementary file 4 — Supplementary Material 4 [file 12872_2023_3602_MOESM4_ESM.png]
